# Improving estimates of global ant biomass and abundance

**DOI:** 10.1073/pnas.2214825119

**Published:** 2022-10-05

**Authors:** Tom M. Fayle, Petr Klimes

**Affiliations:** ^a^School of Biological and Behavioural Sciences, Queen Mary University of London, London, E1 4NS, United Kingdom;; ^b^Biology Centre of the Czech Academy of Sciences, Institute of Entomology, Ceske Budejovice, 37005, Czech Republic

Ants are ubiquitous on Earth, being found in all terrestrial biomes, except for polar regions. Their eusociality, the ability to form mutualistic relationships with a wide range of other organisms, and evolutionarily plastic morphology and diet have made them one of the most successful and ecologically important animal taxa. The late Edward O. Wilson (1) referred to them (among other insects) as “the little things that run the world.” Although global patterns of ant diversity are reasonably well understood (2), there is still a surprisingly high degree of uncertainty regarding how many ant individuals there are in the world and subsequently, the extent to which ants contribute to global biomass. In PNAS, Schultheiss et al. (3) address these knowledge gaps by gathering most of the literature to date on ground-dwelling ant abundances in the leaf litter on Earth. Using this extensive dataset, they scale ant abundance values from over 26,800 m^2^ of leaf litter up to the level of individual biomes and habitat types, resulting in greatly improved estimates for global ant abundance and biomass.

Why do we need global estimates of ant biomass and abundance? Our planet is changing rapidly due to a range of threats, including habitat destruction, hunting, and climate change. This is of concern since changes to the structure of biological communities affect the way that ecosystems function, particularly for groups that mediate multiple important ecosystem processes, such as ants. Although much attention has focused on the influence of species diversity on ecosystem functioning (4), the abundance and biomass of a group of organisms are also important. For example, higher ant abundances are linked to more rapid removal of food resources (5), greater effects on soil properties (6), and stronger influence in food webs (7). Furthermore, some local scale studies indicate that arthropod biomass is decreasing due to anthropogenic influences (8). However, shifts in biomass and abundance of arthropods at global scales remain poorly understood (9). This is of concern, since without good baseline data on abundance and biomass of ants and other ecologically important taxa, future changes will be challenging to quantify. We currently have good estimates of global abundance and biomass of vertebrates (10) but not of terrestrial invertebrates, where the current best estimate of 200 megatonnes of carbon (Mt C) (10) is based on a semiquantitative expert opinion of global arthropod abundance from over 60 y ago (11). Hence, quantification of global ant biomass and abundance has until recently remained an important knowledge gap.

**Fig. 1.**
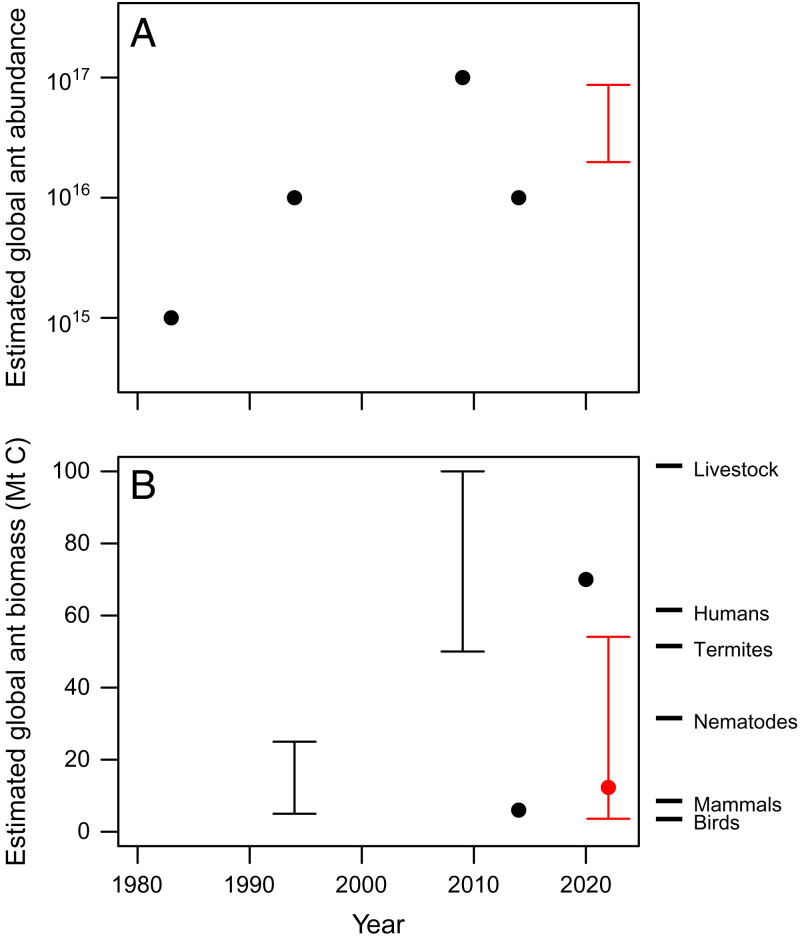
Historical progress in estimating (*A*) global ant abundance and (*B*) global ant biomass. Points represent individual estimates of abundance or biomass. Range bars indicate multiple estimates from a single study under different assumptions. Estimates from Schultheiss et al. (3) are in red, with their best estimate of 12.3 Mt C as a central point and the alternative estimates presented in the study’s supplementary information as range bars. Current best estimates of global biomass for a range of other groups are presented to the right in *B*. Abundances for other groups are not plotted, as they vary too widely to be visualized on the same plot as the ant abundances. Sources for ant estimates by year are 1983 (12), 1994 (13), 2009 (14), 2014 (23), 2020 (15), and 2022 (3). Note that for completeness, we include two estimates from nonpeer-reviewed sources (12, 23). In three cases, it was necessary to convert the numbers into megatonnes of carbon (13, 14, 23). Sources for biomass estimates for other groups are ref. 10 for livestock, humans, wild mammals, and birds; ref. 15 for termites; and ref. 24 for nematodes.

Previous estimates of global abundance and biomass of ants (Fig. 1) have taken a “top-down” approach. Many have been based directly (or indirectly) on an estimate for global insect abundances of 10^18^ (11). However, even the author of that paper suggested that this estimate was probably only correct to two orders of magnitude (11). Intriguingly, the first ever published estimate of global ant abundance of 10^15^ individuals was made in a children’s nonfiction book (12), where no details of calculations were given, although unnamed scientists were consulted. Later peer-reviewed estimates ranged between 10^16^ (13) and 10^17^ (14) ants globally, depending on whether ant individuals were assumed to comprise 1 or 10% of total insect abundance. These figures have led to estimates of global biomass of ants of between 5 and 100 Mt C, with differences relating to both the ant abundance estimate used and the mean weight of carbon assumed for a single ant. All these previous studies heroically extrapolate from very modest datasets to global abundances and biomasses, hence the large variation in numbers reported. Critically, all previous estimates were made by multiplying estimations for global arthropod biomass and density by the proportion of that biomass expected to be ants. However, this approach assumes that the proportion of the arthropod fauna that is ants is uniform globally, and the resulting estimates are also dependent on the accuracy of the preexisting total arthropod biomass estimates.

Schultheiss et al. (3) improve greatly on these previous estimates by using a “bottom-up” approach, in which they collate extensive data from published studies that use two common methods for sampling ants: leaf litter sampling and pitfall trapping. Although extrapolations of total global ant abundance and biomass can only be made using the area-based leaf litter sampling data, activity-density data from pitfall trapping are still important since ant functioning in ecosystems is potentially more strongly correlated with ant activity than ant biomass. In addition, corrections are made for unsampled nonforaging workers and for ants living in forest canopies, neither of which are sampled using these methods. The authors estimate that there are 2 × 10^16^ (20 quadrillion) ants on Earth, meaning that for every human living today, there are an astonishing 2.5 million ants. This is at the upper end of previous global ant abundance estimates (Fig. 1). The highest ant abundances are found in tropical moist forests and tropical savannahs. Extrapolations accounting for these differences between biomes in average biomass per unit area give an estimate of global ant biomass of 12.3 Mt C. This is significantly less than some previous estimates (Fig. 1), which ranged up to 70 (15) or 100 Mt C (14). Schultheiss et al.'s results suggest that the proportion of arthropod biomass made up by ants is less than was previously thought and that global ant biomass is only one-fifth that of humans (14). However, there are three reasons why this does not necessarily mean that ants are less ecologically important than previously thought. First, our understanding of the functional importance of ants comes mainly from observations and experiments at local scales. Second, although biomass is less than previously estimated, abundance, which is potentially even more important for ecosystem function, is greater than previously estimated by most authors (Fig. 1). Third, the estimates by Schultheiss et al. (3) are almost certainly rather conservative, and estimated ant abundance and biomass are likely to increase when more data become available.

How might estimates of global ant biomass and abundance be further refined in the future? Schultheiss et al. (3) used mean worker weight measured from a selection of species for which data are available multiplied by the abundance estimates for each biome to give global biomass. This was necessary because only a minority of studies report masses of ant workers or any trait/taxonomic data from which this could be calculated. However, there is significant variation in body mass between ant taxa (16), which should be accounted for once data are available. Further work also needs to be done to assess the impacts of anthropogenic habitat change on global ant biomass and abundance since 55% of the world’s terrestrial ice-free habitats have been modified by humans (17). The current study demonstrates that ant abundance is generally higher in forested habitats compared with plantations, confirming previous results (e.g., ref. 18). However, if ant ecologists have sampled more frequently in less disturbed habitats, as seems likely, then the biome-level extrapolations conducted that did not account for relative areas of anthropogenically modified habitats might be overestimates of current global ant abundance and biomass. Finally, much more work needs to be conducted to quantify ant communities in less well-sampled subhabitats, such as forest canopies and soils, while also considering brood and reproductive castes, and these data will lead to increases in estimates of global ant abundance and biomass. The authors use a smaller fogging dataset to estimate canopy ant numbers, although it is likely that entirely ground-based fogging greatly underestimates ant abundances. It is notable that the highest fogged ant abundances reported by Schultheiss et al. (3) come from one of the few studies (19) in which fogging was conducted across all canopy layers, which reported 806 ants/m^2^ compared with a weighted mean of 40 ants/m^2^ for ground-based fogging at the same location and 55 ants/m^2^ for all tropical/subtropical fogging studies. A recent study incorporating both soil and litter ants at global scales (20) also indicates that abundances of ants may be significantly higher than reported here [for example, abundance in tropical forests is ∼1,000 m^−2^ compared with 28 to 58 m^−2^ as reported by Schultheiss et al. (3)]. Such data should inform the next round of global estimates of ant biomass and abundance. It is vital that global databases collating information on biological communities, such as those used in this study (21, 22), are maintained and expanded in the future to facilitate this future work. Studies such as those of Schultheiss et al. (3) are vital if we want the little things that run the world to continue their central role in global ecosystems in the face of ongoing anthropogenic impacts.
